# Pericytes and mesenchymal stromal cells converge toward pro-tumor phenotypes in the tumor microenvironment

**DOI:** 10.1007/s12672-026-04790-y

**Published:** 2026-03-11

**Authors:** Enrico La Spina, Cesarina Giallongo, Lucia Longhitano, Jessica Ferrigno, Giuseppe Di Rosa, Vittorio Del Fabro, Deborah Calvo, Sebastiano Giallongo, Davide Romano, Andrea Duminuco, Giuseppe A. Palumbo, Fabio Galvano, Giovanni Li Volti, Ignazio A. Barbagallo, Daniele Tibullo

**Affiliations:** 1 Department of Biomedical and Biotechnological Sciences, University of Catania, Catania, Italy; 2 Department of Medicine and Surgery, University of Enna “Kore”, Enna, Italy; 3 Department of Medical and Surgical Sciences and Advanced Technologies “G.F. Ingrassia”, University of Catania, Catania, 95123 Italy; 4 Princess Nuora Bint, Abdulrahman University, Riyad, Saudi Arabia

**Keywords:** Mesenchymal stromal cells, Pericytes, TME

## Abstract

Mesenchymal Stromal Cells (MSCs) and pericytes, although a minor cellular component of the tumor microenvironment (TME), exert outsized control over cancer progression, metastasis, and therapeutic response across both solid and hematologic malignancies. Once separated by functional and anatomical criteria, Single-cell RNA sequencing (scRNA seq) analyses and context-dependent phenotypic transitions - pericyte-to Cancer-Associated Fibroblasts (CAFs) and MSCs-to-CAFs, now blur these classical distinctions, revealing fluid entities and substantial functional convergence. We synthesize current evidence showing that MSCs and pericytes frequently adopt overlapping pro-angiogenic, immunosuppressive, and pro-invasive states driven by PDGF-B/PDGFRβ, TGF-β, CXCL12, and Notch/ROCK signaling. Across cancers, their roles are multifaceted: in Colorectal Cancer (CRC) from neo-vascularization and Drug Resistance (DR) to blood vessel formation, invasion, and metastatic spread. Moreover, MSCs reinforce immunosuppression, whereas pericyte phenotype switching may sensitize tumors to immunotherapy, thus playing a pivotal role in fibrosis-driven cancer progression. In hematologic malignancies, particularly in the Bone Marrow (BM) niche, MSCs sustain leukemic cell survival and DR. Shared markers and transcriptomic signatures, coupled with striking plasticity, underscore their central role in shaping a pro-tumorigenic milieu. This convergence helps to explain the limits of current approaches—such as anti-VEGF monotherapy and supports new strategies. Enhancing pericyte maturity or intercepting transitions toward CAFs are promising avenues to boost treatment efficacy. We propose a practical framework for classifying “MSC-pericyte states” in the TME and emphasize rigorous, multi-marker, spatially resolved analyses to dissect their complex functions, thus opening a new scenario for targeted therapies.

## Introduction

The TME is a complex and dynamic ecosystem in which malignant cells coexist with a heterogeneous array of stromal, vascular, and immune components that collectively regulate tumor initiation, progression, metastasis, and therapeutic response [1, 2]. Beyond genetic alterations intrinsic to cancer cells, mounting evidence indicates that stromal compartments critically shape tumor behaviour at the tissue level through coordinated regulation of angiogenesis, immune dynamics, extracellular matrix (ECM) remodelling, and therapeutic responsiveness [3]. Among stromal populations, MSCs and pericytes have emerged as key, yet incompletely defined, regulators of the TME across solid and haematological malignancies [4, 5]. MSCs have traditionally been described as multipotent stromal progenitors with immunomodulatory and tissue-supportive functions, whereas pericytes have been classically defined as perivascular mural cells responsible for microvascular stability and barrier regulation [6]. However, these distinctions—largely derived from in vitro criteria, culture-based definitions, and limited marker panels—have proven insufficient to capture their biological complexity in vivo. Developmental studies and single-cell transcriptomic analyses indicate that pericytes are ontogenetically related to, and often considered a vessel-associated subset of, the broader mesenchymal stromal compartment, sharing mesenchymal origin and partially overlapping transcriptional programs with MSCs [5, 7]. At the same time, accumulating in vivo and spatial evidence indicates that pericyte identity is defined primarily by perivascular localization and vascular-supporting functions, rather than by exclusive marker expression, whereas MSCs represent a more heterogeneous stromal pool distributed across interstitial and perivascular niches whose phenotype is strongly shaped by tissue context and microenvironmental cues [8, 9]. Recent advances in scRNA-seq transcriptomics, spatial profiling, and lineage-tracing approaches have revealed extensive heterogeneity and plasticity within both MSCs and pericyte populations, particularly within tumor-associated contexts [4, 10]. These studies indicate that MSCs and pericytes frequently share transcriptional programs, functional outputs, and surface markers, complicating their unambiguous identification and supporting a state-based interpretation of mesenchymal stromal biology, in which stromal populations are better understood as dynamic, context-dependent states rather than rigid lineage-defined entities [10, 11]. Within tumors, MSCs and pericytes are exposed to a constellation of microenvironmental cues—including hypoxia, inflammatory cytokines, aberrant growth factor signalling, mechanical stress, and metabolic alterations—that profoundly reprogram their phenotype and function [12, 13]. As a result, both cell types display pro-angiogenic, immunosuppressive, ECM-remodelling, and therapy-tolerant states that actively support tumor growth and progression [14, 15]. Importantly, this tumor-driven convergence does not negate the existence of distinct physiological identities and homeostatic roles for MSCs and pericytes in non-malignant tissues but rather underscores the capacity of the TME to override baseline constraints and impose shared stress-adaptive programs [16, 17]. Failure to account for this context-dependent plasticity has contributed to inconsistencies in the literature and may partially explain the limited efficacy of therapies targeting stromal or vascular components [18]. In this review, we synthesize current evidence on the physiological roles of MSCs and pericytes and their tumor-associated phenotypic and functional adaptation across solid and haematological malignancies. We emphasize shared stromal programs that shape the TME, while highlighting the limitations of marker-based definitions and the critical importance of spatial and functional context. By integrating these observations, we propose a coherent framework for understanding MSCs–pericyte plasticity in the TME and its implications for stromal-targeted therapeutic strategies.

### Physiological identity versus tumor-reprogrammed states: MSCs and pericytes compared

In physiological conditions, MSCs and pericytes occupy partially overlapping yet functionally distinguishable stromal niches, reflecting their shared mesenchymal origin but divergent anatomical anchoring and baseline roles [5]. MSCs are broadly distributed within interstitial connective tissues and perivascular regions, where they contribute to tissue homeostasis by supporting stem and progenitor cell niches, coordinating wound repair, modulating immune tone, and maintaining ECM turnover [5, 19]. Pericytes, in contrast, are anatomically defined mural cells embedded within the vascular basement membrane and tightly associated with endothelial cells along capillaries and post-capillary venules, where they regulate microvascular stability, barrier integrity, and capillary hemodynamic [4, 6]. Despite these physiological distinctions, MSCs and pericytes share expression of multiple markers—including PDGFRβ, NG2/CSPG4, CD146/MCAM, and αSMA in specific contexts— which may undergo phenotypic interconversion under defined experimental or pathological conditions, thereby limiting the reliability of single-marker–based attribution [4, 20]. Under non-malignant conditions, pericyte identity is primarily defined by spatial localization and vessel-associated functions, whereas MSCs are defined more broadly by stromal niche activities and multipotency-associated programs rather than by fixed anatomical positioning [5, 9]. Within tumors, however, both populations undergo extensive reprogramming toward convergent pro-tumorigenic phenotypes, with fibroblast-like activation, and the establishment of therapy-tolerant stromal niches [6, 15, 21]. This convergence does not negate their physiological distinctions but instead underscores the capacity of tumor-specific cues to override homeostatic constraints and impose shared stress-adaptive transcriptional and functional programs across stromal lineages [12]. A comparative overview of the physiological identity and tumor-associated reprogramming of MSCs and pericytes—encompassing spatial localization, marker expression and functional roles—is summarized in Table 1.

### Tumor-derived cues driving MSC–pericyte convergence

TME exerts strong selective pressures that rewire stromal cell identity and function through the combined action of biochemical, mechanical, and metabolic cues [6, 10]. Hypoxia and aberrant vascular perfusion activate Hypoxia-Inducible Factor (HIF)-dependent transcriptional programs and promote the release of Vascular Endothelial Growth Factor (VEGF) and Platelet-Derived Growth Factor (PDGF)-family ligands, profoundly influencing both MSCs and pericyte behaviour and favouring pathological angiogenesis and vascular dysfunction [13, 22]. Chronic inflammatory signalling further sustains stromal activation, with cytokines such as Transforming Growth Factor-beta (TGF-β), Interleukin-1(IL-1), and Tumor Necrosis Factor (TNF), together with chemokines including C-X-C motif chemokine ligand 12 (CXCL12), promoting immunosuppressive, pro-fibrotic, and immune-excluding phenotypes [12, 19]. In parallel, increased mechanical stress and ECM stiffening enhance mechanotransduction pathways, including RhoA/ROCK signalling, thereby reinforcing contractile, migratory, and fibroblast-like programs in both cell types [1, 6]. Metabolic alterations within tumors—such as lactate accumulation, oxidative stress, and nutrient competition—further bias MSCs and pericytes toward survival-oriented and immune-modulatory outputs [23, 24]. Collectively, these tumor-derived cues drive phenotypic drift and functional convergence toward CAF-like, immunoregulatory, and pro-angiogenic stromal states, providing a unifying mechanistic explanation for the context-dependent efficacy and frequent resistance observed with vascular- and stroma-targeting therapies [14, 18].

**Table 1:** Physiological identity and tumor-associated reprogramming of MSCs and pericytes

Feature	Pericytes	MSCs
Physiological localization	Pericytes are anatomically defined mural cells embedded within the vascular basement membrane and tightly associated with endothelial cells along capillaries and post-capillary venules, where their identity is primarily spatial rather than marker-based [4]	MSCs are broadly distributed within interstitial connective tissues and perivascular niches across organs, lacking a fixed anatomical anchoring and exhibiting context-dependent localization [5]
Physiological functions	In non-malignant tissues, pericytes regulate microvascular stability, endothelial survival, barrier integrity, capillary hemodynamic, and tissue perfusion, contributing to vascular quiescence	MSCs support tissue homeostasis by maintaining stem/progenitor niches, coordinating wound repair, modulating immune tone, and regulating ECM turnover [5]
Lineage and conceptual definition	Pericytes are considered vascular mural cells of mesenchymal origin, with heterogeneous developmental lineages depending on tissue and embryonic origin [5]	MSCs represent a heterogeneous population of mesenchymal stromal progenitors defined operationally by functional plasticity rather than a single lineage or anatomical position [9]
Commonly used markers	Pericytes commonly express PDGFRβ, NG2/CSPG4, CD146/MCAM, desmin, and αSMA in a context- and activation-dependent manner[6]	MSCs are typically identified by expression of CD73, CD90, and CD105 according to ISCT criteria, with frequent expression of PDGFRβ depending on tissue and state [21]
Marker overlap and limitations	Marker expression alone is insufficient to define pericyte identity, as substantial overlap exists with MSCs and CAFs, necessitating spatial and functional validation [20]	MSC marker expression significantly overlaps with pericytes and CAFs, and in vitro marker-based definitions often fail to reflect in vivo tumor-associated states[21]
Tumor-derived reprogramming cues	Within tumors, pericytes are reprogrammed by hypoxia, aberrant PDGF-B/PDGFRβ and VEGF signalling, inflammatory cytokines, mechanical stress, and metabolic alterations [13]	Tumor-associated MSCs are shaped by hypoxia, TGF-β signalling, inflammatory cytokines, chemokines, oxidative stress, and metabolic competition [12]
Functional outputs in the TME	Tumor-reprogrammed pericytes contribute to pathological angiogenesis, vascular abnormality, immune modulation, and therapy resistance, including regulation of drug delivery and immune cell trafficking [14]	MSCs in the TME adopt CAF-like, immunosuppressive, and pro-invasive programs, supporting tumor growth, immune exclusion, and resistance to chemotherapy and immunotherapy [15]
Plasticity and convergence	Pericytes exhibit high plasticity and transition toward fibroblast-like or inflammatory stromal states under tumor pressure, contributing to functional convergence with MSCs [20]	MSCs display marked phenotypic plasticity and differentiate toward CAF-like or pericyte-like states depending on microenvironmental cues [12]
Key experimental caveats	Pericyte identification requires integration of spatial localization, multimarker panels, and functional assays, as αSMA expression is highly state-dependent [4]	MSC identification should avoid reliance on culture-based criteria alone and incorporate spatial, functional, and transcriptomic information [21]

## Solid tumor: shared MSC–pericyte stromal programs

### Angiogenesis and perivascular dysfunction

Across solid tumors, MSCs and pericytes converge toward pro-angiogenic and perivascular signalling programs that profoundly alter vascular architecture, function, and spatial organization within the TME [6, 13]. Aberrant activation of PDGF-B/PDGFRβ and VEGF pathways drives pathological angiogenesis, vessel instability, and impaired perfusion, thereby promoting hypoxia and downstream DR [18, 22]. ScRNA-seq analyses across multiple tumor types have revealed that pericytes and perivascular MSCs-like populations do not constitute a homogeneous compartment but instead segregate into transcriptionally distinct states characterized by variable expression of angiogenic, contractile, metabolic, and inflammatory programs, often correlating with their spatial proximity to tumor vessels and hypoxic regions [24]. Experimental evidence from in vivo tumor models and ex vivo vascular assays further indicates that alterations in pericyte recruitment, coverage, and activation state lead to abnormal vessel morphology, increased vascular leakage, and heterogeneous perfusion patterns, emphasizing that vascular dysfunction is tightly linked to the spatial arrangement and transcriptional state of perivascular cells rather than vessel density alone [18, 24]. In CRC, in vitro co-culture systems and orthotopic xenograft models demonstrated that pericytes coordinate endothelial remodelling and spatially restricted crosstalk with cancer stem cell niches, sustaining neovascularization and facilitating metastatic dissemination [19]. ScRNA profiling of CRC supports these observations by identifying perivascular stromal clusters enriched for PDGFRβ, ECM-remodelling genes, and pro-angiogenic signatures, spatially localized at invasive fronts and perivascular regions [10, 25]. In glioblastoma (GBM), lineage-tracing and in vivo transplantation studies have shown that Glioma Stem Cells (GSCs) generate tumor-associated pericyte-like cells that integrate into the perivascular niche, where their close spatial proximity to endothelial cells reinforces vascular support functions and sustains tumor growth [26]. In breast cancer, spatial profiling and scRNA analyses revealed marked heterogeneity in pericyte coverage and transcriptional states across tumor regions, with poorly covered or transcriptionally activated perivascular zones displaying vascular instability and increased metastatic potential [27, 28]. Experimental modulation of PDGF-B signaling redistributes pericyte positioning along tumor vessels, resulting in partial vessel normalization and improved chemosensitivity in preclinical models [18, 27]. In melanoma, functional studies in syngeneic models combined with scRNA analyses demonstrate that pericyte-dependent regulation of vascular barrier properties and immune cell trafficking is spatially restricted to defined perivascular niches, linking pericyte localization and transcriptional state to immune exclusion and therapeutic responsiveness [14, 17]. In Hepatocellular Carcinoma (HCC), scRNA-seq identified activated Hepatic Stellate Cells (HSCs) as dominant perivascular stromal populations exhibiting fibrogenic, angiogenic, and immunomodulatory transcriptional programs These liver-resident pericytes respond to PDGF and TGF-β signaling by spatially coupling angiogenic remodeling with ECM deposition [29, 30].

### Immune modulation

MSCs and pericytes shape immune landscapes in solid tumors by adopting immunoregulatory phenotypes that restrict immune cell infiltration and function [12, 17]. CXCL12-centered chemokine programs, adhesion modules, and cytokine signalling promote immune exclusion and recruitment/maintenance of suppressive myeloid populations [12, 30]. More recently, single-cell RNA sequencing has provided direct evidence that these immunomodulatory properties correspond to distinct stromal transcriptional states rather than uniform cell identities. Pan-cancer and tumor-specific scRNA-seq datasets identified stromal clusters expressing CXCL12, C-C motif chemokine ligand 2 (CCL2), and immune checkpoint–modulating ligands that overlap transcriptionally with both mesenchymal and perivascular signatures, supporting the existence of shared MSCs–pericyte immunoregulatory programs [10]. In melanoma, MSCs-driven immunosuppression has been shown to limit the efficacy of immune checkpoint blockade, whereas pericyte phenotype switching alleviates immune exclusion and sensitizes tumors to immunotherapy in preclinical models [14, 31]. In GBM, pericytes act as active immune-regulatory hubs whose functions vary with tumor proximity and contribute to local immunoregulatory control within the brain TME [32]. Spatially resolved scRNA-seq and transcriptomic profiling in GBM further revealed that pericytes form localized immune-regulatory hubs, with transcriptional programs that vary according to proximity to tumor cells and vasculature, directly shaping immune cell exclusion within the brain TME [32].In breast and CRC, stromal remodelling and perivascular signalling reshape immune surveillance and associate with therapy-response modulation [25, 33]. In HCC, activated HSCs modulate the immune microenvironment, promoting immune evasion programs in fibrosis-linked HCC progression [34, 35].

### ECM remodelling, fibrosis, and CAF-like transition

A hallmark of MSCs–pericyte convergence in solid tumors is transition toward CAF-like, ECM-remodelling states that promote tissue stiffening and invasive growth [15, 20]. Recent scRNA-seq analyses demonstrated that CAFs populations arise from multiple stromal sources and exist as dynamic functional states rather than fixed differentiation endpoints [10, 11]. In a comprehensive CAFs classification framework, Zhang et al. [11], identified immunoregulatory and myofibroblastic CAF programs that transcriptionally overlap with mesenchymal and perivascular signatures, supporting the concept that MSCs and pericytes serve as functional precursors of CAF-like states under tumor-driven stress and inflammatory cues. Tumor cues such as TGF-β, inflammatory signalling, and mechanotransduction, including RhoA/ROCK-linked programs, promote perivascular-to-interstitial drift and myofibroblast-like activation [1, 12]. In CRC and breast cancers, MSC-to-CAF programs enhance invasion, metastasis, and DR through ECM deposition and stromal stiffening [36, 37]. In HCC carcinoma, hepatic stellate cells (liver-specific pericytes represent a dominant fibrogenic engine that couples fibrosis to tumor progression and immune remodelling [34, 35]. In GBM, perivascular stromal/pericyte programs contribute to niche architecture that supports invasion and therapy adaptation, consistent with a broader CAF-like functional drift under tumor pressure [26, 38]. In melanoma, stromal education by MSC secretomes can promote EMT-like/invasive programs in tumor cells, fitting with ECM-remodelling and invasion-permissive stromal states [39].

### Therapy resistance

Therapy resistance emerges as a recurrent functional outcome of MSCs–pericyte reprogramming, arising from the integration of spatial, metabolic, and paracrine mechanisms within the TME [14, 40]. Perivascular localization, altered perfusion, metabolic rewiring, and paracrine signalling collectively limit drug delivery and promote tumor cell survival under treatment pressure [24]. In GBM, pericyte-derived CCL5–CCR5 signalling increases Temozolomide resistance by enhancing DNA damage repair within the perivascular niche [40]. In engineered and in vivo tumor models, pericyte–tumor cell paracrine crosstalk has been shown to modulate drug sensitivity through signalling networks that intersect metabolic rewiring and survival pathways, reinforcing therapy-tolerant niche functions [41]. In CRC, tumor-educated MSCs can drive CAF-like programs associated with invasion and DR through ICAM1/STAT3/AKT-linked signalling and stromal reprogramming [36]. In melanoma, stromal immunosuppression and myeloid-mediated resistance are central barriers to ICI efficacy, and vascular/perivascular programs can modulate these resistance states [14, 30]. In breast cancer, vessel normalization and pericyte-related modulation impact chemosensitivity and metastatic progression, linking perivascular biology to treatment failure or response depending on context [18]. In liver cancer, the fibrosis-conditioned TME and stromal programs contribute to therapeutic resistance landscapes and limited efficacy of systemic treatments [42].

## Haematological malignancies: MSC–pericyte programs in the BM niche

### Mesenchymal stromal niche support of leukemic cell survival

In haematological malignancies, MSCs and perivascular stromal cells form a core component of the BM niche that regulates both normal haematopoiesis and malignant cell fate [43, 44]. Tumor-educated MSCs provide survival cues, cytokines, metabolic support, and adhesion-mediated protection that sustain leukemic proliferation/dormancy and therapy tolerance [45, 46]. In Acute Myeloid Leukemia (AML) and B-cell Acute Lymphoblastic Leukemia (B-ALL), MSC-mediated niche protection reduces chemotherapy efficacy and promotes disease persistence [43, 45]. In Chronic Myeloid Leukemia (CML), microenvironmental programs contribute to drug tolerance, and targeting stress-adaptive pathways such as hemeoxigenase-1 (HO-1) has been proposed to overcome resistance mechanisms in leukemic settings [47]. In Myeloproliferative Neoplasms (MPNs), MSCs-driven remodelling and fibrotic reprogramming create a permissive niche that sustains malignant haematopoiesis and disease progression [48].

### Inflammation, oxidative stress, and fibrotic remodelling

Chronic inflammation and oxidative stress reshape the BM microenvironment in myeloid malignancies, driving MSCs activation and fibrotic remodelling [48, 49]. In MPNs, PDGF-related signalling, inflammasome activity, and redox imbalance promote fibrosis-linked stromal dysfunction and reinforce malignant niche features [49, 50]. In AML, mesenchymal niche remodelling programs have been shown to impair haematopoiesis and support leukemic evolution through stromal reprogramming mechanisms [51]. In B-ALL, MSC-supported niches reinforce leukemic survival and can participate in chronic pro-survival inflammatory signalling loops within the marrow ecosystem [45]. In CML, redox/stress-response pathways (including HO-1–linked programs) intersect with DR biology and stromal protection concepts in leukemic systems [47]. These inflammation/fibrosis programs mirror CAF-like transitions in solid tumors, supporting the concept of shared stromal stress-adaptive states across cancer contexts [20].

### Vascular and perivascular regulation of the BM niche

Pericytes and perivascular stromal cells maintain BM vascular integrity and regulate trafficking between blood and marrow compartments, influencing oxygen/nutrient gradients and cell positioning [44, 51]. In AML, stromal and vascular niche remodelling alters microenvironmental constraints that regulate hematopoietic outputs and may favour leukemic stem/progenitor persistence [51]. In B-ALL, perivascular MSC niches are central to leukemic maintenance and spatial protection within the marrow [45]. In MPNs, fibrotic remodelling impacts vascular–stromal coupling and is tightly linked to disease-associated niche dysfunction [48]. In CML, vascular/perivascular niche protection is conceptually consistent with therapy-tolerant microenvironments that limit eradication despite kinase inhibition [47]. More broadly, dynamic changes of MSC progeny and perivascular niche components during leukemia support the view that perivascular dysfunction contributes to abnormal marrow architecture in haematological cancers [52].

### Immunomodulation and therapy tolerance

MSCs and stromal niche cells exert potent immunosuppressive effects in haematological malignancies by shaping myeloid suppressor networks, T-cell dysfunction, and cytokine circuits [52, 53]. In AML, stromal remodelling and inflammatory niche programs support immune evasion and therapy tolerance features in the marrow ecosystem [51]. In B-ALL, MSC-supported niches sustain leukemic persistence and can contribute to immunoregulatory/protective microenvironments that reduce treatment efficacy [45]. In MPNs, inflammasome-linked inflammation and redox imbalance reinforce immunomodulatory and fibrosis-associated stromal programs that sustain disease evolution [49, 50]. In CML, microenvironment-linked survival programs are consistent with therapy tolerance mechanisms that complicate durable eradication despite targeted therapy [47]. Collectively, these processes establish therapy-tolerant BM niches that mirror, in a tissue-specific way, the stromal protection observed in solid tumors [44].

## A practical framework for MSC–pericyte states in the TME

### Perivascular functional states: vessel-supporting to angiogenic-reactive continuum

Perivascular MSCs and pericytes comprise stromal populations that remain spatially associated with the vasculature and exhibit a continuum of functional activation states ranging from vessel-stabilizing to angiogenic-reactive phenotypes [4]. At the homeostatic end of this continuum, cells are tightly embedded within the vascular basement membrane and closely opposed to endothelial cells, expressing PDGFRβ and NG2/CSPG4 with low inflammatory and ECM–remodeling signatures, thereby supporting barrier integrity, endothelial survival, and controlled regulation of microvascular tone [6, 54]. Under tumor-derived cues such as hypoxia, aberrant perfusion, and dysregulated PDGF/VEGF signalling, these perivascular cells transition toward an angiogenic-reactive state while maintaining vessel association [13, 22]. In this context, activation of PDGFRβ-driven pathways and a pro-angiogenic secretome enhances crosstalk with endothelial cells, contributing to pathological angiogenesis and an unstable balance between vessel normalization and vascular abnormality, with direct consequences for perfusion and therapeutic delivery [18]. To integrate these observations across cancer contexts, we propose an operational framework of MSC–pericyte functional states in the TME, as summarized in Table 2. Importantly, experimental evidence that pericyte depletion can accelerate tumor progression indicates that loss of vessel-stabilizing perivascular states may itself be tumor-promoting, underscoring that pericyte functions are context- and state-dependent rather than uniformly pro-tumorigenic [55].

### Stress-adaptive stromal states: CAF-like remodelling and immunoregulatory convergence

MSC–pericyte plasticity also gives rise to stress-adaptive stromal phenotypes that are less strictly perivascular and increasingly shaped by inflammatory, mechanical, and metabolic pressures within the TME [18, 20]. Accordingly, MSC-mediated effects may range from tissue-protective and anti-inflammatory in specific contexts to pro-tumorigenic under chronic stress and inflammatory signalling, highlighting the importance of distinguishing physiological versus tumor-imposed stromal states. In these conditions, both MSCs and pericytes can migrate from perivascular to interstitial compartments and adopt CAF-like myofibroblastic programs driven by TGF-β signalling, mechanotransduction pathways including RhoA/ROCK, and chronic inflammatory cues [1, 12]. These phenotypic transitions promote ECM deposition, tissue stiffening, invasion tracks, and fibrosis-linked tumor progression, thereby reinforcing physical and biochemical barriers to effective therapy [37]. Within this broader stress-adaptive spectrum, immunoregulatory stromal programs frequently emerge, characterized by CXCL12-centered chemokine networks and adhesion modules that regulate immune cell trafficking and positioning [12, 17]. Functionally, these programs foster immune exclusion, recruitment of myeloid-derived suppressor cells and tumor-associated macrophages, and reduced responsiveness to immune checkpoint blockade, although experimental evidence indicates that phenotype switching remains reversible in selected contexts [12, 30].

### Operational criteria and therapeutic implications

Assignment of MSC–pericyte states should not rely on single markers but instead integrate multimarker expression patterns, spatial localization, and functional readouts [4]. Spatially resolved approaches, including spatial transcriptomics, spatial proteomics, and multiplex immunofluorescence, are essential to anchor pericyte identity by anatomical context and to distinguish perivascular from interstitial stromal states [10]. Functional assays assessing endothelial stabilization, barrier support, ECM contraction, and immune-cell trafficking are required to assign biologically meaningful stromal phenotypes [6]. From a translational perspective, therapeutic strategies may aim to reinforce vessel-stabilizing perivascular states to improve perfusion and drug delivery or to intercept transitions toward CAF-like and immunosuppressive stromal programs through context-dependent targeting of PDGFRβ, TGF-β, or ROCK signalling pathways, thereby enhancing the efficacy of chemotherapy and immunotherapy [14, 18, 56]. From a translational perspective, several challenges must be considered when targeting MSC–pericyte stromal programs. Although multiple therapeutic strategies aimed at PDGFRβ signalling, TGF-β pathways, vascular normalization, or stromal contractility have entered clinical evaluation, their efficacy has been variable, in part due to the functional plasticity and context dependence of stromal states [57]. Emerging evidence suggests that stromal features such as pericyte coverage, spatial proximity to tumor vessels, and CXCL12-rich niches may function as predictive biomarkers of therapeutic response, particularly for anti-angiogenic and immunotherapeutic approaches [27]. However, targeting stromal populations remains challenging because disruption of vessel-stabilizing or homeostatic pericyte states can paradoxically accelerate tumor progression, while stromal cells can dynamically switch phenotypes under treatment pressure [8, 55]. These observations underscore the need for biomarker-guided, state-specific stromal targeting strategies rather than blanket depletion approaches and highlight the importance of longitudinal and spatially resolved analyses in clinical trial design [8, 55].

**Table 2:** Operational framework of MSC–pericyte states in the TME

MSC–Pericyte State	Molecular / multimarker features	Spatial criteria	Dominant functional outputs
Perivascular contractile /vessel-supporting state	This state is characterized by expression of PDGFRβ and NG2/CSPG4 with low inflammatory and ECM-remodelling signatures, reflecting a contractile but quiescent transcriptional profile [4]	Cells are tightly embedded within the vascular basement membrane and in direct contact with endothelial cells, defining pericyte identity primarily by spatial localization [6]	Supports vascular stability, endothelial survival, barrier integrity, and controlled regulation of microvascular tone, contributing to normalized perfusion [54]
Activated angiogenic / perivascular signalling state	This state shows activation of PDGFRβ/PDGF and VEGF-related signalling pathways, with a pro-angiogenic secretome and enhanced endothelial crosstalk [13]	Cells remain perivascular but display a reactive phenotype associated with abnormal or remodelling vessels [22]	Drives pathological angiogenesis, vessel instability, and context-dependent balance between vascular normalization and dysfunction [18]
CAF-like myofibroblastic / ECM-remodelling state (MSC–pericyte convergence	This state is associated with subset-dependent αSMA/ACTA2 expression, PDGFRβ signalling, and robust ECM gene programs, though none of these markers are exclusive [20]	Cells frequently transition from perivascular to interstitial locations, losing strict vascular anchoring during tumor progression [15]	Promotes collagen deposition, tissue stiffening, invasion tracks, fibrosis-linked tumor progression, and therapy resistance [37]
Immunoregulatory / chemokine-rich stromal state	This state is defined by CXCL12-centered chemokine programs and expression of adhesion and immune-modulatory molecules regulating leukocyte trafficking [12]	Cells localize in perivascular and/or interstitial niches adjacent to immune infiltrates and tumor-associated vasculature [17]	Mediates immune exclusion, recruitment of MDSCs and TAMs, and reduced efficacy of immune checkpoint inhibitors, although phenotype switching can sensitize tumors in specific contexts [14, 30]
Therapy-resistant niche-supporting state	This state integrates stress-adaptive transcriptional programs linked to hypoxia, metabolic rewiring, and DNA damage tolerance [24]	Typically enriched in perivascular or hypoxic niches that shelter tumor cells from therapeutic exposure [51]	Supports tumor cell survival, stemness maintenance, and resistance to chemotherapy and targeted therapies [24]

### Single-cell and spatial atlases defining MSC–pericyte heterogeneity and functional states

Recent advances in scRNA-seq, single-nucleus RNA sequencing, and spatial transcriptomic approaches have provided unprecedented resolution of mesenchymal and perivascular heterogeneity in human tumors. Pan-cancer scRNA-seq demonstrated that stromal compartments are composed of multiple, dynamically interconverting mesenchymal states rather than discrete, lineage-fixed cell types. Notably, the pan-cancer analysis by Luo et al. [10], identified conserved CAF programs across solid tumors that overlap transcriptionally with MSC- and pericyte-associated signatures, supporting the concept of shared stress-adaptive mesenchymal states rather than strictly segregated populations. Tumor-specific scRNA-seq studies have further refined this view by resolving perivascular and mesenchymal heterogeneity within defined anatomical niches. In GBM, combined scRNA-seq with functional analyses identified pericyte subsets that form therapy-resistant perivascular niches and actively modulate tumor cell survival [11]. Complementary work by Ngo and colleagues [38] demonstrated that distinct perivascular stromal states regulate invasion and treatment response, underscoring the functional specialization of MSC–pericyte–like populations within the vascular niche. In breast cancer, scRNA and spatial profiling have revealed substantial heterogeneity in perivascular coverage and mesenchymal programs across molecular subtypes, while earlier integrative analyses by Kim et al. [27]. connected pericyte heterogeneity with metastatic propensity and therapeutic response. Liver cancer studies have highlighted the close relationship between fibrosis, stellate cell activation, and pericyte-like programs. ScRNA-seq of HCC, identified activated HSCs as dominant fibrogenic and immunomodulatory stromal populations, reinforcing the notion that liver-resident pericytes represent a tissue-specific manifestation of broader MSC–pericyte plasticity [10]. Beyond solid tumors, scRNA profiling of the BM niche has provided critical insights into stromal remodelling in haematological malignancies demonstrated that leukemic stress reprograms MSC and perivascular niches, impairing normal haematopoiesis and supporting malignant persistence [51]. Collectively, these datasets illustrate that MSC and pericyte identities are best understood as context-dependent stromal states defined by spatial localization, transcriptional programs, and functional outputs rather than rigid cell-type boundaries. Key scRNA-seq and spatial transcriptomic resources that have informed current concepts of MSC–pericyte plasticity and convergence across tumor types are summarized in Table 3.

**Table 3:** Representative ScRNA-seq and spatial transcriptomic studies defining MSC and pericyte heterogeneity in human tumors

Cancer type	Citation	Title		Key MSC–pericyte insight
Pan-cancer	[10]	*Pan-cancer scRNA-seq analysis reveals the heterogeneity and plasticity of cancer-associated fibroblasts in the TME*	scRNA-seq	Identifies conserved CAF/mesenchymal states across tumors overlapping with MSC/pericyte programs
GBM	[40]	*Pericytes augment glioblastoma cell resistance to TMZ through CCL5–CCR5 paracrine signalling*	scRNA-seq + functional assays	Defines pericyte subsets forming therapy-resistant perivascular niches
GBM	[38]	*Perivascular stromal cells instruct glioblastoma invasion, proliferation, and therapeutic response*	scRNA-seq + spatial analysis	Identifies functionally distinct perivascular stromal states regulating invasion and treatment response
Breast cancer	[28]	*ScRNA-seq analyses reveal evolution mimicry during the specification of breast cancer subtype*	scRNA-seq	Resolves stromal and pericyte-like heterogeneity linked to subtype evolution
Breast cancer	[27]	*Heterogeneous perivascular cell coverage affects breast cancer metastasis and response to chemotherapy*	scRNA-seq + spatial profiling	Links pericyte heterogeneity to metastasis and chemosensitivity
HCC	[10]	*ScRNA-seq of the TME in HCC*	scRNA-seq	Identifies activated stellate/pericyte-like fibrogenic states
Haematological malignancies (AML)	[51]	*Mesenchymal niche remodelling impairs haematopoiesis *via* stanniocalcin-1 in AML*	scRNA-seq	Reveals leukemic reprogramming of MSC/perivascular niches

## Discussion

A growing body of evidence indicates that pericytes are not only vascular support cells but can also directly influence tumor cell fitness through paracrine communication. Pericyte-derived secreted factors can activate tumor cell stress-adaptive programs, enhancing survival under treatment pressure and promoting resistance to chemotherapy and targeted agents [41, 58]. Taken together, the preceding sections support a state-based view of MSCs and pericytes as dynamic regulators of tumor ecosystems rather than passive structural elements. Across cancer contexts, both populations display marked plasticity and partial functional convergence, frequently adopting overlapping programs that promote pathological angiogenesis, immune exclusion, ECM remodelling, invasion, and therapy tolerance [2, 6, 16]. In this framework, the classical distinction—MSCs as multipotent stromal progenitors versus pericytes as vessel-associated mural cells—remains a useful starting point, but it becomes insufficient when tumor-imposed cues reshape phenotype, transcriptional identity, and function in vivo [5, 15]. Consistently, ScRNA-seq and spatial profiling, together with experimentally observed transitions such as pericyte-to-CAF and MSC-to-CAF routes, argue that stromal biology is better understood as a spectrum of context-dependent states rather than rigid lineage-fixed cell types [20, 59, 60]. Mechanistically, convergence is repeatedly driven by a limited set of recurrent axes—PDGF-B/PDGFRβ, TGF-β, CXCL12-centered chemokine programs, and mechanotransduction modules (including ROCK-linked pathways)—that collectively “educate” mesenchymal and perivascular compartments toward pro-tumor outputs [23]. Tumor-derived PDGF-B can potentiate MSC–pericyte transition and recruitment in experimental settings [13], illustrating how growth factor signalling can override homeostatic constraints and enforce shared stress-adaptive programs. Importantly, “plasticity” in this context should not be interpreted as a single phenomenon: it includes (i) functional reprogramming without lineage change (e.g., acquisition of inflammatory, immunoregulatory, or pro-angiogenic secretomes), and (ii) fate-like transitions or positional drift (e.g., perivascular-to-interstitial movement with CAF-like activation), which together blur boundaries between MSC, pericyte, and CAF-like phenotypes [20, 59, 60]. This distinction is essential for interpreting marker overlap and for designing experimental readouts that are spatially and functionally anchored. A first major consequence of MSC–pericyte state switching is vascular dysfunction. Aberrant PDGF/VEGF signalling and hypoxia-associated programs can destabilize vessel structure, impair perfusion, and exacerbate hypoxia, thereby reinforcing selection for aggressive tumor behaviour and limiting therapeutic delivery [13, 18, 22]. At the same time, perivascular states are not uniformly pro-tumorigenic: evidence that pericyte depletion can accelerate tumor progression and disrupt barrier function underscores that loss of vessel-stabilizing states may itself be tumor-promoting and that “pericyte targeting” must be state-specific rather than indiscriminate [32, 61]. This duality directly informs the translational strategy: restoring or maintaining vessel-supporting perivascular states can, in principle, improve perfusion and drug penetration, whereas blanket depletion risks worsening hypoxia and invasiveness. A second key consequence is immune remodelling and immune exclusion. MSC–pericyte–like stromal programs frequently converge on CXCL12-centered chemokine networks, adhesion modules, and cytokine signalling that restrict effector infiltration, spatially segregate immune cells, and promote suppressive myeloid circuits [14, 30, 31]. Preclinical observations that pericyte phenotype switching can alleviate immunosuppression and sensitize tumors to immunotherapy highlight that stromal states can be reversible in selected contexts, supporting “re-education” approaches over depletion [14]. These immunoregulatory features are also consistent with the broader concept that perivascular and stromal niches can function as immune checkpoints at the tissue level, shaping how immune cells traffic and persist within tumors [14, 30]. A third recurrent outcome is CAF-like activation and ECM remodelling. Under TGF-β signalling, inflammatory cues, and mechanical stress, both MSCs and pericytes shift toward myofibroblast-like programs, increasing collagen deposition, stiffening, and invasion-permissive tracks [1, 8, 28]. These ECM changes are not merely structural: they alter mechanotransduction, growth factor availability, and immune-cell access, thereby linking fibrosis-like remodelling to both invasiveness and therapy resistance [15, 20]. This helps reconcile why similar stromal pathways recur across organ-specific settings, even when the resident stromal populations differ (e.g., hepatic stellate cells as liver-specific pericyte-like fibrogenic effectors) [34, 35, 42, 62]. Finally, stromal states contribute to therapy tolerance through combined physical, metabolic, and signalling mechanisms. Perivascular localization, altered perfusion, hypoxia-driven stress programs, and paracrine signalling collectively promote tumor cell survival under treatment pressure [24, 40]. In this landscape, stromal protection is not limited to structural “shielding”: pericyte–tumor paracrine crosstalk can directly modulate drug sensitivity by engaging survival and stress-adaptation pathways [41, 58]. These concepts extend naturally to haematological malignancies, where MSC-driven niche protection is a well-established determinant of leukemic persistence and therapy resistance [45, 46, 53], and where perivascular elements and vascular remodelling can influence trafficking, gradients, and fibrosis-linked niche dysfunction [51, 52]. From a translational perspective, these observations argue against non-selective disruption of stromal or perivascular compartments. Clinical experiences have already suggested that combined pathway inhibition can be limited by toxicity and by incomplete efficacy when stromal states adapt or compensate; for example, PDGFRβ inhibition combined with VEGF inhibition did not provide benefit and increased toxicity in renal carcinoma [24]. Instead, a more plausible strategy is biomarker-guided, state-specific intervention: reinforcing vessel-supporting states to improve delivery, intercepting CAF-like and immunoregulatory drift, or reprogramming stromal phenotypes to restore immune accessibility. Preclinical work supports the feasibility of stromal re-education, including pericyte phenotype switching associated with vessel normalization and improved anti-tumor immunity [14, 63]. However, the field must also explicitly incorporate contradictory findings and tissue-specific exceptions, including settings where stromal programs may be protective or where perturbation of homeostatic pericyte functions accelerates disease [9, 32, 61, 64–66]. Overall, the most consistent path forward is not “targeting MSCs or pericytes,” but targeting the states they adopt—defined by multimarker features, spatial context, and functional outputs—using longitudinal and spatially resolved analyses to capture treatment-induced switching and to avoid counterproductive interventions.

## Conclusion

MSCs and pericytes represent a central, state-dynamic stromal axis in the TME. Across solid tumors and haematological malignancies, their partially overlapping identities, pronounced plasticity, and functional convergence toward angiogenic, immunoregulatory, ECM-remodelling, and therapy-tolerant programs shape tumor progression, metastatic potential, and resistance to treatment [31, 67–69]. A key message emerging from current evidence is that these populations cannot be reliably defined by single markers or culture-derived criteria. Instead, accurate attribution requires integrated approaches that combine multimarker panels, spatial localization (particularly perivascular embedding), ScRNA-seq and spatial transcriptomic signatures, and functional validation. Future progress will depend on experimentally grounded, state-based classification frameworks that distinguish fate transitions from functional activation shifts and capture reversible versus stable stromal programs. Integrating longitudinal ScRNA-seq and spatial profiling with lineage tracing and perturbation studies will be critical to map upstream drivers, downstream effectors, and treatment-induced state changes. Ultimately, deciphering MSC–pericyte heterogeneity as operational “states” rather than rigid cell types should enable biomarker-guided, context-specific stromal interventions that improve drug delivery, reverse immune exclusion, and reduce therapy resistance—thereby enhancing the efficacy of existing regimens and supporting more precise, personalized strategies for aggressive malignancies.

## Data Availability

No datasets were generated or analysed during the current study.

## Abbreviations

AKT: Protein Kinase B
AML: Acute Myeloid Leukemia
B-ALL: B-cell Acute Lymphoblastic Leukemia
BM: Bone marrow
CAF/CAFs: Cancer-Associated Fibroblasts
CCR5: C-C Motif Chemokine Receptor 5
CML: Chronic Myeloid Leukemia
CRC: Colorectal Cancer
CSCs: Cancer Stem Cells
CXCL12: C-X-C Motif Chemokine Ligand 12
DR: Drug Resistance
ECM: Extracellular Matrix
GBM: Glioblastoma Multiforme
GSCs: Glioma Stem Cells
HCC: Hepatocellular Carcinoma
HSCs: Hepatic Stellate Cells
ICAM1: Intercellular Adhesion Molecule 1
ICIs: Immune Checkpoint Inhibitor
MDS: Myelodysplastic Syndrome
MDSCs: Myeloid-Derived Suppressor Cells
MPN / MPNs: Myeloproliferative Neoplasms
MSCs: Mesenchymal Stromal Cells
PDGF-B: Platelet-Derived Growth Factor-B
PDGFRβ: Platelet-Derived Growth Factor Receptor-Beta
ROCK: Rho-Associated Protein Kinase
ScRNA-seq: Single-cell RNA sequencing
STAT3: Signal Transducer and Activator of Transcription 3
TGF-β: Transforming Growth Factor-Beta
TME: Tumor microenvironment
VEGF: Vascular Endothelial Growth Factor
